# Development and Health of Adults Formerly Placed in Infant Care Institutions – Study Protocol of the LifeStories Project

**DOI:** 10.3389/fnhum.2020.611691

**Published:** 2021-01-20

**Authors:** Patricia Lannen, Hannah Sand, Fabio Sticca, Ivan Ruiz Gallego, Clara Bombach, Heidi Simoni, Flavia M. Wehrle, Oskar G. Jenni

**Affiliations:** ^1^Marie Meierhofer Children’s Institute, University of Zurich, Zurich, Switzerland; ^2^Child Development Center, University Children’s Hospital Zurich, Zurich, Switzerland

**Keywords:** longitudinal study, lifespan development, institutional care, adverse childhood experiences, early childhood, child development, compulsory social measures and placements

## Abstract

A growing volume of research from global data demonstrates that institutional care under conditions of deprivation is profoundly damaging to children, particularly during the critical early years of development. However, how these individuals develop over a life course remains unclear. This study uses data from a survey on the health and development of 420 children mostly under the age of three, placed in 12 infant care institutions between 1958 and 1961 in Zurich, Switzerland. The children exhibited significant delays in cognitive, social, and motor development in the first years of life. Moreover, a follow-up of a subsample of 143 children about 10 years later revealed persistent difficulties, including depression, school related-problems, and stereotypies. Between 2019 and 2021, these formerly institutionalized study participants were located through the Swiss population registry and invited to participate once again in the research project. Now in their early sixties, they are studied for their health, further development, and life-course trajectories. A mixed-methods approach using questionnaires, neuropsychological assessments, and narrative biographical interviews was implemented by a multidisciplinary team. Combining prospective and retrospective data with standardized quantitative and biographical qualitative data allows a rich reconstruction of life histories. The availability of a community sample from the same geographic location, the 1954–1961 cohort of the Zurich Longitudinal Studies, described in detail in a paper in this issue (Wehrle et al., 2020), enables comparison with an unaffected cohort. This article describes the study design and study participants in detail and discusses the potential and limitations of a comparison with a community sample. It outlines a set of challenges and solutions encountered in the process of a lifespan longitudinal study from early childhood into the cusp of old age with a potentially vulnerable sample and summarizes the lessons learned along the way.

## Introduction

In Switzerland, placing infants in institutions was not uncommon in the first half of the 20th century (Ryffel, 2013). The main reasons for having a child placed in an institution were either being an unmarried or underaged mother or having a migrant background, foremost Italian migrant worker status (German: *Gastarbeiter*) (Meierhofer and Keller, 1974). Having a child as a young, unmarried mother was, from the point of view of the authorities and society, slovenly (liederlich) and was to be “disciplined” (Ramsauer, 2000; Lengwiler and Praz, 2018; Unabhängige Expertenkommission Administrative Versorgungen, 2019). Migrant workers were subjected to serious prejudice and residence permit restrictions and were forced to work full time with long working hours to stay in Switzerland (D’Amato, 2012; Joris, 2012). Generally, infants were placed into institutions at a very young age, before the age of two weeks, due to the lack of paid maternity leave (Huber, 1995). At that time, the infant was seen as a simple reflex-driven being (Meierhofer, 1958), and a belief was prevalent that “there will be no harm to infants if they are cared for by strangers” (Meierhofer and Keller, 1974). Hard-earned success in reducing child mortality had made preventing the spread of germs a priority, so an institutional practice of “isolation” was the norm, involving as little physical contact as possible, feeding according to a rigid plan, and strict hygiene (Ryffel, 2013). Care practices were generally characterized by strict routines that did not take into consideration individual needs and an intense wariness of spoiling children (Gebhardt, 2009). This created the conditions of chronic deprivation found to be responsible for the profound negative effects on development described in more recent work (Nelson et al., 2014; Berens and Nelson, 2015).

A growing volume of international research shows that children placed in institutional care as it is commonly implemented are typically deprived of a supportive, intensive, one-to-one relationship with a primary caregiver. Such conditions of deprivation are profoundly damaging to children, particularly during the critical early years of development (Schore, 2001). Children who were placed into institutions shortly after birth and subjected to severe sensory, emotional, and sometimes physical neglect show a dramatic decrease in brain activity compared to children who were never institutionalized (Center on the Developing Child, 2007). They are more likely to suffer from growth delay, frequent infections, and hearing and vision problems. Furthermore, motor development is often delayed and stereotypical behaviors such as body rocking and head banging occur (Browne, 2009; Berens and Nelson, 2015; Sherr et al., 2017). Finally, a number of studies have found negative effects on cognitive and social development (Schore, 2001; Johnson et al., 2006). Although no data is currently available on the effects of institutionalization across the entire lifespan, a number of large studies have demonstrated the potential long-term negative effects of other adverse events and circumstances during childhood, such as child abuse and neglect, household substance abuse, and mental illness, on development and health into adulthood (Felitti et al., 1998; Werner, 2013). The impact of such events has also been shown to be especially severe during the critical developmental stages in early childhood, with possible permanent effects on the morphology of the brain (Shonkoff and Phillips, 2000).

However, not all individuals experience negative health outcomes in relation to stress. In his seminal work, Antonovsky (1979) coined the term “salutogenesis” after observing “how people manage stress and stay well.” Similarly, findings from a 40-year longitudinal study by Werner (2013) showed that one third of all high-risk children exposed to adverse experiences early on displayed resilience that allowed them to develop into caring, competent, and confident adults. Particular protective factors and biographical events helped to balance out risk factors at critical periods in their development in a dynamic interaction of personal factors, environmental factors, and biographical developments. Further, evidence has started to emerge of what mitigates the negative impact of institutionalization, such as age at entry, stability of care, size of institution and children/staff ratio (Berens and Nelson, 2015; Sherr et al., 2017). Care circumstances and practices with children in institutions can vary greatly, with some of the most severe conditions of deprivation studied as part of the Bucharest Early Intervention Study with children in Romania (Nelson et al., 2014). This study was also able to show that when children are moved from institutional care into family based care, they have a chance of restoring brain development, highlighting the plasticity of the developing brain (Nelson et al., 2014; Bick et al., 2015).

The potential impact of this early institutionalization across the lifespan is unknown. Furthermore, whereas data is available on the effects of exposure to adversity in early childhood into early and mid-adulthood, the effects of adverse experiences into late adulthood remain as yet virtually unexplored. With a unique combination of historical and newly collected 60-year long-term follow-up data, the overall aim of the LifeStories project (German: *Lebensgeschichten*) is to examine the personal developmental trajectories of individuals that were affected by placement in institutions as infants in the late 1950s and early 1960s in Switzerland. The study builds on the pioneering work of Dr. Marie Meierhofer on the delayed development of infants placed in institutions for care at the end of the 1950s (Meierhofer and Keller, 1974). By simultaneously using data from an unaffected comparison sample of children growing up in families, the 1954–1961 cohort of the Zurich Longitudinal Studies of the University Children’s Hospital Zurich (ZLS, *N* = 445) —one of the most significant data sets on child development globally (Ulijaszek et al., 1998)—the project will shed light on the impact of certain institutional care practices during infancy across the lifespan.

## Methods and Analysis

### Design

**FIGURE 1:**
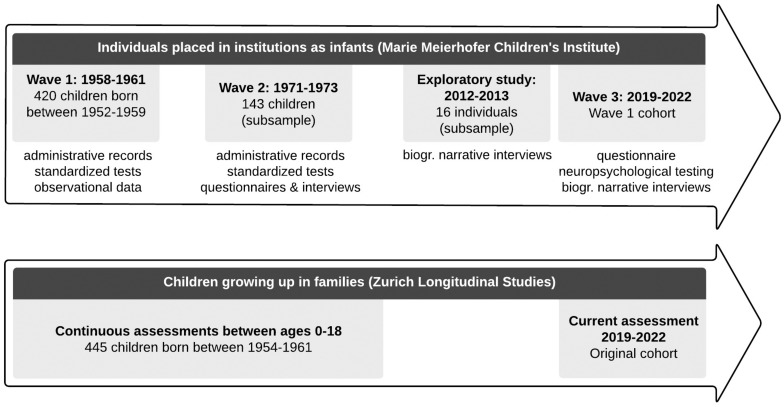
Study design.

This population-based study uses a combined prospective and retrospective, mixed-methods approach over a 60-year period to investigate how the lives of individuals who had been placed in an institution as infants in the late 1950s in Switzerland developed subsequently. The availability of data from the 1954–1961 cohort of the Zurich Longitudinal Studies enables comparison with an unaffected group of children growing up in families at the same time and in the same geographic region. The overall study design is depicted in Figure 1. This article describes the cohort of children placed in institutions as infants. The ZLS are described in the paper in this issue (Wehrle et al., 2020).

The following sections describe the assessment at three time points of the cohort of infants placed in institutions.

#### Wave 1: 1958–1961

**FIGURE 2:**
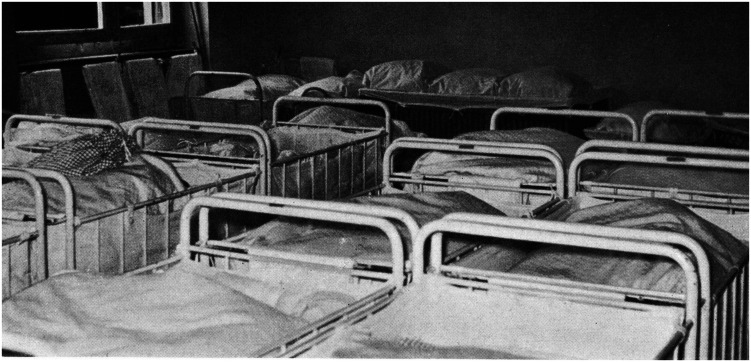
Example of one of the rooms infants were kept in at one of the 12 institutions (Meierhofer and Keller, 1974).

As a baseline, this project uses data from the study conducted by Dr. Meierhofer between 1958 and 1961. She conducted a survey on health and development in 12 infant and toddler care institutions in Zurich, Switzerland. Data collection took place over 16 months. During this period, around 630 children were placed in these 12 institutions. Figure 2 provides an example of an infant room in one of these institutions to illustrate the living conditions at that time. Children who fulfilled the following eligibility criteria were included in the study: children were at least three months old at the time of the examination, they were not older than six months at the first time of in-care-placement, their institutional stay was never interrupted for more than three months, and they did not have any diagnosed medical disorder. In addition, children were excluded if they suffered from any acute infectious disease at the time of the examination or responded with distress to the test situation. This resulted in a sample of *N* = 429.^1^.

Two types of data were collected during Wave 1: person-centered data for each child and institution-centered data for each institution in which children were placed. Data was collected as a mixed-method assessment and included quantitative and qualitative data.

##### Person-centered data

For all children, demographic data, the psychosocial situation of the family, nature and frequency of current contact with the family, and information on children’s institutional stay (e.g., age at in-care placement) were recorded. Because in most instances it was not possible to obtain this information directly from the parents, Dr. Meierhofer used the administrative records available (e.g., guardianship records) and information provided by staff members of the care institutions. These data sources were also used to record children’s health status (e.g., previous diseases, birth and pregnancy history). Some of this information was recorded in standardized, self-developed study templates; other data was documented in narrative notes and underwent quantitative coding afterward. To assess children’s development, a standardized developmental test was used [*Échelle de développement*, Brunet-Lézine for all children up to 2.7 years (Brunet and Lézine, 1955), Terman-Merrill developmental test for children up to 4.6 years (Terman and Merrill, 1937), and *Schweizertest* for children up to 7.6 years (Biäsch and Fischer, 1969)]. The individual test result was used to calculate an overall developmental quotient (DQ) for each child. Furthermore, height and weight were measured. In addition, children’s behavior and interaction with staff members, other children, and the examiner were assessed by observations during daily routines in the institutions and during the test situation. In general, the observational data was collected and documented in a qualitative manner, and some of the data was subsequently quantitatively coded. In addition, original photographs, slides, and film has been preserved.

##### Institution-centered data

Information on childcare circumstances and practices in the different institutions was collected, such as interaction time, child-staff-ratio, and the educational background of staff members. In addition to information provided by the heads of the institutions, observations were recorded by the researchers during daily routines (see Table 1 for an overview of the instruments used at Wave 1).

**TABLE 1:** Overview of the assessment instruments (Wave 1 and 2).

Wave	Type of data	Instrument (References)	Subconstruct
1	Administrative records and information provided by staff members of the care institutions	n.a.	Demographic Variables/Family background
			Contact with the family
			Information on institutional stay
			Health status
			Childcare circumstances and practices (on institutional level)
	Standardized tests	Brunet–Lézine (Brunet and Lézine, 1955)	Overall development (children up to 2.7 years)
		Termann–Merill (Terman and Merrill, 1937)	Overall development (children up to 4.6 years)
		Schweizertest (Biäsch and Fischer, 1969)	Overall development (children up to 7.6 years)
		n.a.	Body Weight and Height
	Observational data	n.a. (part of the data scored according to Thalmann’s symptom-burden scale, Thalmann, 1971)	Behavior
			Interaction
			Childcare circumstances and practices (on institutional level)
2	Administrative records and information provided by parents/caregivers	n.a.	Demographic Variables/Family background
			Care history
			Academic career
			Health status and pubertal development
	Standardized tests/questionnaires	WIP (Dahl, 1972)	Cognitive abilities
		Kinder-Angst-Test (Thurner and Tewes, 1969)	Mental health
		Rorschach-Test (Bohm, 1965)	Personality
		Foto-Hand-Test (Belschner et al., 1971)	Aggressive behavior
		Sohnaufsatz (Ungricht, 1955)	Graphology
		Baumtest (Koch, 1957)	Character/affective development
		Sociogram (Bastin, 1967)	Social status
		Polaritätenprofil (Instrument developed for the present study)	Personality, learning and academic behavior
		n.a.	Body Weight and Height
	Semi-structured interviews	n.a. (part of the data scored according to Thalmann’s symptom-burden scale, Thalmann, 1971)	Care histories, development, social relationships, and education

Today, most of the data from Wave 1 is stored in the Federal Archive in Aarau. The research team has been granted permission to access and analyze this historical data for scientific purposes related to the historic reappraisal of care practices before 1981, on condition that it is used in anonymized form, according to article 11c of the Swiss Federal Act on the Reappraisal of Compulsory Social Measures and Placements before 1981 (German: *Bundesgesetz über die Aufarbeitung der fürsorgerischen Zwangsmassnahmen und Fremdplatzierungen vor 1981*) and the Act on Public Information, Data Protection and Archives (German: *Gesetz über die Information der Öffentlichkeit, den Datenschutz und das Archivwesen; IDAG*) and the corresponding by-law (VIDAG).

Unfortunately, even though additional data from Wave 1 was retrieved from various private archives as well as the archive of the Marie Meierhofer Children’s Institute, some of the data of this initial assessment remains missing. However, identifying data has been preserved for 98% of the original cohort: for 420 of the 429 children. Aggregated developmental data (e.g., DQ) is available for the majority of the children up to 3 years at the time of Wave 1 (*n* = 322).

**FIGURE 3:**
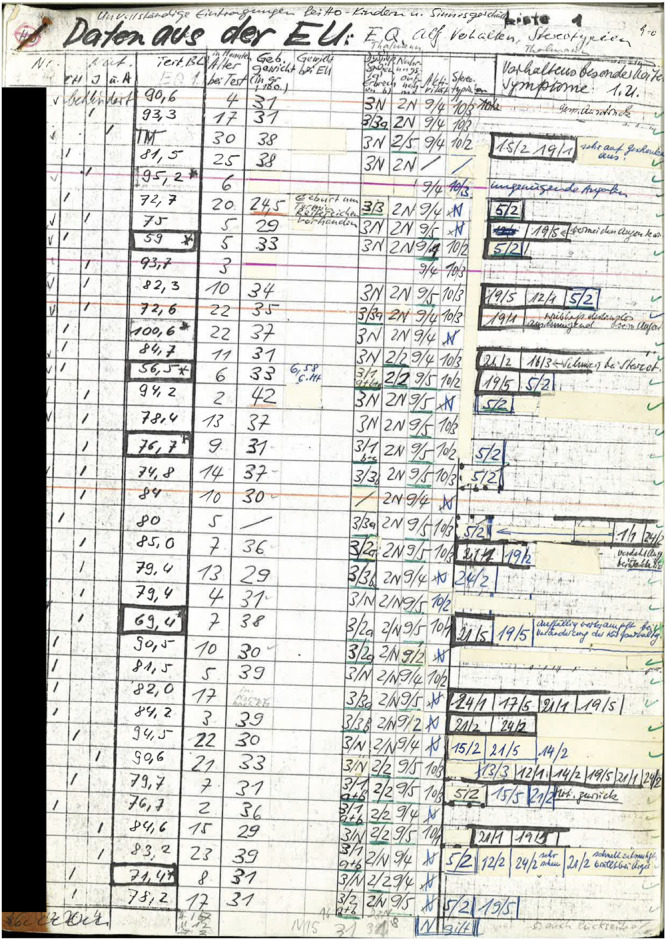
Example of person-centered data (Wave 1).

To make this data accessible, all documents were retrieved from the archives as scans or digital photographs. Because this data is only available in analog form (mostly hand-written, some of it typed up with manual typewriters), data had to be entered manually into electronic form. A couple of issues posed a challenge to systematic data entry: firstly, information for the individual children was not available in a systematically compiled form but was distributed over many different datasheets. Secondly, the information was not recorded consistently across all of the 12 institutions but was collected using many slightly different datasheets (see Figure 3 for an example of the available data). To deal with this and to reduce errors during data entry, standardized input templates were created for quantitative data (Limesurvey GmgH, 2020). Data was then imported into R (R Core Team, 2018) and merged for further processing and analysis. Qualitative data was typed up in Microsoft Word. Photographs, audio files, and film have been digitized for preservation.

In her analyses of the data at the time, Dr. Meierhofer found that the children placed in institutions had significant delays in cognitive, social, and motor development compared to the children growing up in families studied by the ZLS. Differences in children’s development within the group of institutionalized children could not be explained by families’ socio-economic status or the infants’ contact with their family members. However, differences in the quality of care (i.e., interaction time and child–staff ratio) between the 12 institutions included in the study accounted for the variance in children’s development. Meierhofer and Keller (1974) concluded that the developmental delays of the children were primarily caused by the poor conditions of care in the institutions.

#### Wave 2: 1971–1973

To assess the developmental progress and health status of the children, Dr. Meierhofer and her team conducted a follow-up study of a subsample of the children between 1971 and 1973, then aged between 13 and 15 years (Meierhofer and Hüttenmoser, unpublished).

Of the original cohort, Dr. Meierhofer only considered children born between 1957 and 1959, who were up to age three years at the time of Wave 1, for the follow-up study (*N* = 354). The address for 82 of the eligible children was not found at the time, and so it was not possible to contact them. An additional 13 children were excluded as they had moved to other parts of Switzerland and the distance from these children was deemed too great for their inclusion in the study. For 64 children, parents or legal guardians actively declined consent for participation. Another 24 did not respond to the invitation to participate (presumed passive decline). Two of the children had died. An additional ten children were excluded for other reasons (not further specified). Of the remaining 159 children, 16 participated in a preliminary study between 1969 and 1971 (Meyer-Schell, 1971)^2^ and were then excluded from further study. The final sample of the full follow-up study therefore consisted of *N* = 143 children. The distribution of gender, nationality, and marital status of the mother within the group of children examined was, according to Meierhofer and Hüttenmoser (unpublished) approximately comparable to that of the overall sample. In the further course of the study, 17 of these children with suspected epilepsy or other neurological disorders were excluded from further analyses (Meierhofer and Hüttenmoser, unpublished).

Data collection during Wave 2 was carried out with a multi-method and multi-informant approach: quantitative and qualitative data were collected using administrative records, standardized tests and questionnaires, and interviews with parents or other primary caregivers, teachers, and the children themselves.

To obtain general information on the family background, children’s care histories (e.g., number of different placements, reasons for placement changes), and their academic career, the study team used administrative data available, for example, from guardianship records. If necessary, parents or other caregivers were asked to provide further information. Most of the information was qualitative in nature and documented using standardized templates developed by the study team^3^.

Standardized testing was conducted using a battery of neuropsychological tests and questionnaires: Children’s cognitive abilities were assessed with the WIP, a short version of the Hamburg Wechsler Intelligence test for children (Dahl, 1968, 1972). Their mental health was assessed using the *Kinder-Angst-Test* (KAT), (Thurner and Tewes, 1969), a standardized questionnaire on children’s anxiety. Furthermore, two projective tests were conducted: the Rorschach-Test (Bohm, 1965) to assess personality and the *Foto-Hand-Test* (Belschner et al., 1971) to assess aggressive behavior. The test battery also included the *Sohnaufsatz* (Ungricht, 1955), in which children had to write a short essay that was later analyzed for both content and graphology, and the *Baumtest* (Koch, 1957), which was intended to provide information on the child’s character and affective development. In addition, children’s height and weight were measured. Furthermore, data on pubertal development and general health was collected through information provided by the parents or caregivers. In addition, the social status of the children among their classmates was determined with a sociogram (Bastin, 1967): the children themselves and five of their classmates were asked to indicate with which children they would most like to spend time and with whom they would least like to spend time. The number of children’s mutual choices was evaluated.

In addition, semi-structured interviews were conducted with primary caregivers, children themselves, and their teachers. For the interviews with the parents, key themes were predefined, such as pregnancy and birth history, care histories, children’s development (motor skills, language, sleep, tidiness), social relationships, and education. Furthermore, interviews with the children contained questions about school, leisure activities, friendships, and their ideas about their future. The teacher survey focused on academic performance and social contact with classmates. If necessary, interviews with migrant workers were conducted in Italian and subsequently translated. The original audio-recordings and some of the transcripts have been preserved. The qualitative information collected during the interviews was scored according to Thalmann’s symptom-burden scale (Thalmann, 1971) and used to assess children’s behavior. In addition, teachers and researchers recorded information on the children’s personality and learning and academic behavior (for example reliability, discipline, independence, pace of work) on a 5-point standardized scale (*Polaritätenprofil*). Table 1 provides an overview of all instruments used at Wave 2.

At the time of Wave 2, data from the ZLS was not ready for comparison. Dr. Meierhofer and her team therefore selected the instruments for the follow-up-study so that data of a normative sample and, if possible, even comparative data from Switzerland was available that allowed basic comparisons (Meierhofer and Hüttenmoser, unpublished).

In her analysis, Dr. Meierhofer found that at the time of Wave 2, children who were placed in institutions as infants showed increased depression, school related-problems (e.g., significantly higher grade retention rate than the comparison group), and stereotypies (Meierhofer and Hüttenmoser, unpublished). Due to a fierce nature/nurture debate with one of her colleagues at the time, these results were never published (Wyss-Wanner, 2000) and exist only in an original research report in the archives of the Marie Meierhofer Children’s Institute (Meierhofer and Hüttenmoser, unpublished).

**FIGURE 4:**
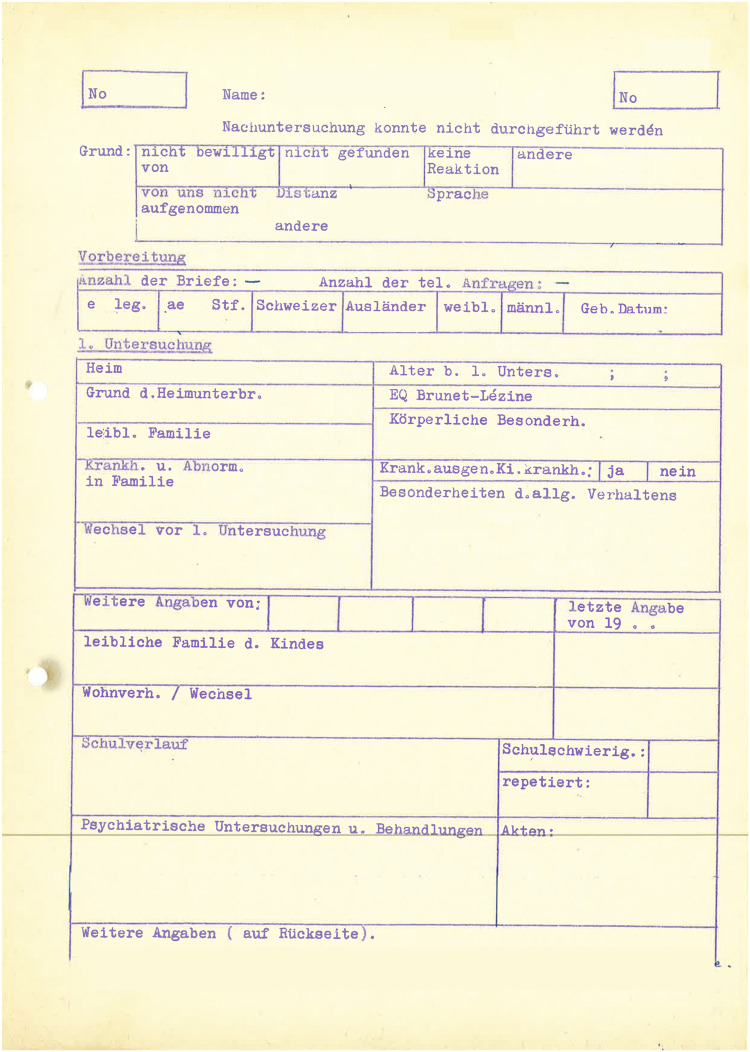
Example of person-centered data (Wave 2).

In contrast to the Wave 1 data, the raw Wave 2 data has been fully preserved. Part of the data is stored in paper form at the Federal Archive in Aarau. It has been digitized in the same way as the Wave 1 data by first retrieving the data from the archive as digital photographs and then entering the data manually into data entry masks created with LimeSurvey (Limesurvey GmbH, 2020). The remaining data is available in paper form and on microfilm in the archive of the Marie Meierhofer Children’s Institute and has now also been digitized for further processing. Figure 4 provides an example of the available data. Just as for the Wave 1 data described above, data entry masks were created in LimeSurvey (Limesurvey GmbH, 2020), and further data processing were carried out with R (R Core Team, 2018).

Qualitative data will be typed up in Microsoft Word either from available audio files or from original transcripts.

#### Wave 3: 2019–2021

In a newly revived effort funded under the umbrella of the Swiss National Research Program 76, which is focused on scientific investigation into the compulsory social measures and placements before 1981, all individuals that took part in the study by Dr. Meierhofer at Wave 1 were once again located and contacted. These individuals were about 60 years when taking part in this third assessment of health and development (Wave 3).

##### Study preparations

Prior to Wave 3, two preparatory studies were conducted. They are separate, small studies but are briefly summarized here.

###### Exploratory study

Between 2012 and 2013, an exploratory study was implemented. Semi-structured narrative interviews were conducted with 16 individuals that had taken part in Waves 1 and 2. As part of the semi-structured interviews, participants talked about topics such as family, institution, foster or adoptive family, relationships, health, education, work life, hobbies, and aging (Ryffel and Simoni, 2016). Furthermore, findings suggested that despite clear indications of resilience in some individuals, many generally struggled to find a sense of belonging and coherence in their lives. This seemed to be the case more often for individuals placed in institutions as a result of having been born to unmarried and/or underaged mothers than born to migrant workers. Children of migrant workers tended to reassured themselves that they were loved by their parents and that it was external circumstances that led to the placement. They remember it as a measure that “made sense” (Ryffel and Simoni, 2016; Sand and Gruber, 2017).

The experience of this exploratory study informed the search strategy, contact procedures, ethical considerations, and research questions for Wave 3 (for details see corresponding sections below). It also reaffirmed the need for a comprehensive long-term follow-up assessment, as it revealed how diverse the life trajectories can be within this cohort. It also showed that individuals are willing to participate in a research study and talk about their biographical trajectories.

###### Participatory research preparation

To prepare for Wave 3, in 2019, we conducted focused interviews with four individuals that had been placed in institutions as children to elicit feedback on procedures, documents, and assessment instruments to be used for contacting the cohort. This was in response to the request of those affected by the compulsory social measures and placements before 1981 to be included in research related to the reappraisal and reconciliation process (UEK Administrative Versorgungen, 2019; Unabhängige Expertenkommission Administrative Versorgungen, 2019). Interviewees’ feedback helped to make documents more understandable and identified wording that might cause insecurities or trigger negative reactions. They also indicated shortcomings in some of the questionnaire items that they thought were potentially misleading or not appropriate for the situation of the participants (Lannen et al., 2020). In addition, they made significant contributions to how best to approach and work with the cohort. This participatory preparation showed that the inclusion of formerly institutionalized individuals in research is feasible and provides substantial benefits to the research quality on historic compulsory social measures and placements (Lannen et al., 2020).

##### Eligibility

Eligibility criteria were defined separately for (a) locating individuals and (b) contacting them.

In order to be eligible for the search, individuals had to have taken part in Dr. Meierhofer’s study at Wave 1 and to have been assigned a study number for which identifying data was available. In addition, a minimum of three data points needed to be available for the search (full name, date of birth, and a location of residence from some point in their lives). A number of individuals were found to have moved abroad (25%, *n* = 107). If they were found to be residing in a country other than their country of origin, we were able to search them through the embassy or the Bureau of Foreign Affairs in charge. If a city they have moved to abroad is known, they can be searched for through the local population registry, if one exists. No search strategy exists for individuals who moved to their country of origin without any indication of location. In this case, individuals abroad are ineligible for the search.

A person who was not found and those deceased were obviously deemed ineligible for contact. Those that were found, were only eligible for contact, if there was no indication that they might have been adopted without their knowledge (happened early in life, was followed by a name change). This was based on the ethical concern of uncovering a potentially unknown adoption and causing distress to the individual. If there was an indication in the historical files that the individual knew about the adoption, or that the adoption happened within the family (e.g., by the new partner of the mother), individuals remained eligible for the study. In addition, individuals also became ineligible for contact if they had a data protection barrier with the municipalities (see below section “Locating individuals”). Finally, individuals who had actively declined study participation when contacted for the exploratory study (Ryffel and Simoni, 2016) were also deemed ineligible.

##### Locating individuals

The search process took place between October 2018 and March 2021.

Due to the sensitive nature of the request, the process of locating individuals after several decades was set up in a way that minimized the risk of false identification of individuals. Therefore, even though very labor intensive, the search for each individual’s identity needed to be officially confirmed through the population registry. Luckily, this was possible, as in Switzerland, every individual is formally registered with the municipality where he or she resides. Individuals who relocate must give notice of departure with the old municipality and formally register with the new municipality, so individuals can be tracked through the system over time (municipal population registry). In addition, events (such as birth, marriage, divorce, or death) are registered in the civil population registry (*Zivilstandsregister*) in a person’s commune of origin (*Heimatort*)^4^.

In Switzerland, legislation differs slightly between the cantons^5^, but generally, municipalities will provide full name, address, and dates of arrival in and departure from the municipality from the population registry to private individuals and organizations acting in the public interest without requiring a reason for the request^6^. If a “credible argument” is made, the municipality that the person moved to and from, date of birth, gender, marital status, and commune of origin (*Heimatort*) can also be released^7^. Before the information is released, the municipality is to verify that the request is made in the public interest^8^; if this is not case, the municipality is not allowed to release the information. Individuals that have actively instated a data protection barrier are excluded^9^. The municipalities also provide information on deceased individuals should the applicant make explicit an interest for the information^10^. Some municipalities requested more detailed information on the public interest of the request. In these cases, detailed information on the study and on data protection was provided. The study was described as a general survey on health and development and the fact that the individuals in question were placed in institutions as infants was not disclosed to protect their privacy.

A successful search for an individual through municipal population registries requires three data points: the full name, date of birth, and a municipality in which the individual has lived at some point. For a majority of individuals that took part in Wave 1, information on municipalities was available through the study archives. For some, we were able to gain access to municipalities of residence through the Zurich’s City and Federal Archive (archived municipal registration files before 1976, archived supplementary files of birth registries, guardianship records, infant care institution files etc.). The municipal authorities released the current address if the individuals still lived in that municipality or released the name of the municipality the person moved to. The address information request was then iterated through as many municipalities as necessary until the current address was found.

For some cases, instead of a municipality of residence, the person’s commune of origin (*Heimatort*) was available through the archives. A formal research request was submitted to the office of civil registry in Zurich and granted under Art. 60 of the *Zivilstandsverordnung vom 28.04.2004* (ZStV; SR 21 1.112.2) to inquire whether a place of residence can be found in the files for individuals still alive or a date of death for those deceased. Upon the precedent of Zurich, civil registries in other locations provided access to the information. For these individuals, the last known address through civil registries was thus identified and then the above-mentioned process of locating individuals through municipalities repeated for these individuals. An additional research request granted access to the information on deceased individuals whether death was of natural or unnatural (accident, crime, or suicide) causes. This information was made available by the civil registries in the municipalities where individuals resided at the time of death.

For a few individuals, their municipality of residence was found through moneyhouse.ch, an online platform providing information drawing among others from the cantonal commercial registers (since full name and date of birth was available through this portal) and then verified with the municipality.

Overall, the procedure for contacting individuals was very resource intensive and is still ongoing. The search was reiterated through up to 10 municipalities until a person’s current address was identified. More than 300 municipalities were contacted, as well as more than 40 offices of civil registries in Switzerland alone. In total, more than 2000 emails were sent.

So far, 86% (*n* = 268 of total 313) of individuals residing in Switzerland were found, out of those, 78% (*N* = 208) were eligible for contact (not deceased or ineligible).

So far, 25% (*n* = 107) of individuals were found to have moved abroad. If a person had moved to a country other than their home country, we placed an inquiry with the embassy or the Bureau of Foreign Affairs. If the person was registered by their country as living abroad, he or she could be located. If a person was not registered but the municipality the person moved to was known, rather than just the country, then analogous to the search in Switzerland, individuals were searched for through the population registry (if some form of population registry existed in that country). This search strategy via municipalities was applied for all individuals who moved abroad regardless of their nationality or home country. Using these strategies, we have identified 47%, *n* = 50 individuals eligible for search abroad. Out of those, we have been able to locate and contact 24% (*n* = 12) so far.

**FIGURE 5:**
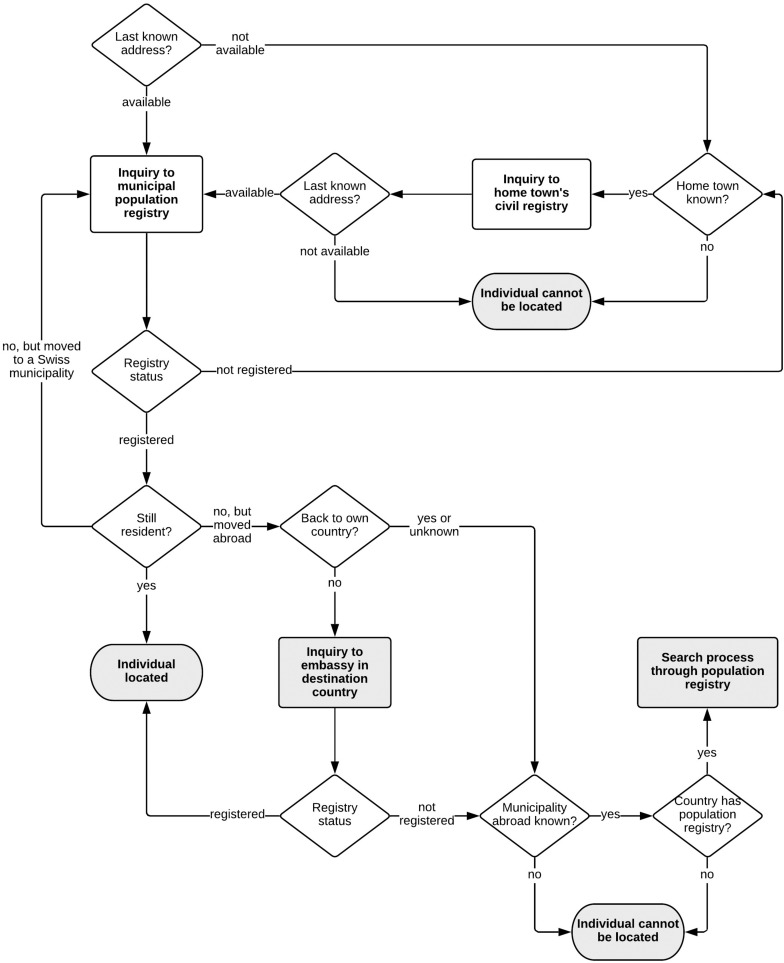
Search process to locate individuals.

Figure 5 shows the search path.

### Contact Procedures

The contact procedure for this study was built on a number of key principles: first, it takes a stepwise approach, so that those individuals that do not wish to learn about any study details can opt out swiftly (ethical protocol, see section “Ethics”). Second, contact procedures were also staggered to ensure sufficient resources were available within the research team for personal contact with the cohort and data collection. Third, contact procedures were designed for a broad range of life trajectories, building on Antonovsky’s salutogenetic approach (Antonovsky, 1987). This is expressed in the wording of documents and behavior of the researchers. Fourth, even though some participants might not remember that they had participated in the study in the past, overall, the study invitation has been framed as an invitation to remain in the study. Finally, the procedure has been set up as an opt-out procedure: at each step, the next point of contact through the research team will be announced, and individuals have to actively let the research team know if they want to disengage from the process. Passive decline has been clearly operationalized as no more contact after a final reminder for participation by mail if a participant cannot be reached by phone after three attempts.

Furthermore, the Swiss Federal Act on the Reappraisal of Compulsory Social Measures and Placements before 1981 (German: *Bundesgesetz über die Aufarbeitung der fürsorgerischen Zwangsmassnahmen und Fremdplatzierungen vor 1981*) stipulates that individuals have the right to view their archived files. Accordingly, the study team has created a leaflet with detailed information on how individuals can access any archived information, either related to their institutional placement or the study.

As an initial form of contact, a short letter with basic information about the study and the individual’s name and year of birth found in the archives was sent to each prospective participant. It also included a request to let the researchers know by phone, email, or returning a slip (a stamped return envelope was included) if they did not want to participate. In that case, they were not contacted again. Two weeks later, anyone who had not actively declined was sent a second letter. This time, more detailed information was provided about the study, including a detailed leaflet that outlined its history, aims, and procedure. It reiterated that participation is voluntary and that they can withdraw at any time. It also outlined data protection measures. In addition, the leaflet specified the components of the study (questionnaire, neuropsychological assessment, biographical narrative interview; for details see section “Data collection”), including expected duration and reimbursement. Individuals received CHF 80. – each for filling in the questionnaire and participating in the neuropsychological assessment, and CHF 40. – for participating in the biographical narrative interview. In addition, they received reimbursement for travel expenses. Again, if anyone indicated that they did not want to participate further (active decline), they were not contacted again. Otherwise, senior staff members called all the individuals for whom we were able to obtain a phone number to invite them to participate in the study and go over the consent form initially over the phone. Notes of the conversation were recorded. Those individuals for whom phone numbers were not available were invited to provide one with a return slip. It was also possible to indicate preferences for communication (mail only, for example, for those who do not want to talk on the phone but would like to participate in the study).

The same researcher remained available throughout the study to ensure consistency and to build trust. If an individual agreed to participate, the questionnaire was sent together with the consent form, and appointments for neuropsychological assessment and interviews were arranged. Help with completing questionnaires was offered when necessary over the phone as a standardized interview. A reminder call was made the day before the appointment. If necessary (e.g., due to poor health), the researcher offered to travel to the participants’ home for data collection.

If the full questionnaire was not returned after 3–4 weeks, the researcher checked in with the participant either by phone or letter to remind the participant to fill in the questionnaire and to see if any assistance was needed. Those individuals who could not be reached by phone were sent a final reminder letter, including the short version of the questionnaire, a consent form, and a slip to indicate active decline or interest in further participation. Sending a final reminder letter with a questionnaire is based on the work of the *Zürcher Längsschnittstudie Von der Schulzeit bis ins mittlere Erwachsenenalter* (Schallberger and Spiess Huldi, 2001), which led to a substantial number of additional questionnaires returned.

For those individuals that were contacted as part of the exploratory study (Ryffel and Simoni, 2016), letters were tailored to the individuals’ responses.

**FIGURE 6:**
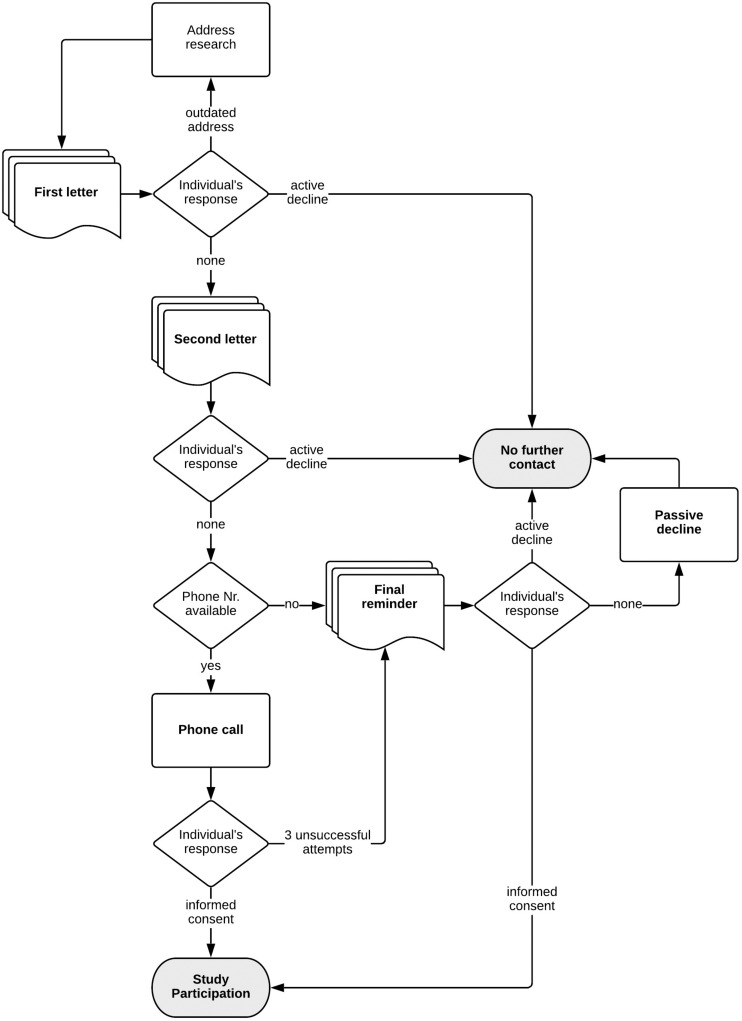
Recruitment process.

The recruitment process is depicted in Figure 6.

#### Data collection

Data collection started in September of 2019, is still ongoing, and is expected to last throughout Q1 of 2021.

A mixed-methods approach was chosen to maximize depth and breadth of data and to enable the accommodation of data collection to individuals’ possibilities and preferences where necessary. Specifically, three types of data were collected: questionnaire data, data from neuropsychological assessments, and data from biographical narrative interviews. The full battery of questionnaire data covered physical and mental health, social abilities and demographics, information on work and family life, retrospectively captured education and professional background, critical life events, and transitions. It included constructs such as sense of coherence, self-efficacy, and the ability to fulfill one’s basic psychological needs, which are all factors believed to be important moderators for adjustment and fulfilled, happy lives. All constructs and the corresponding operationalization and instruments used to assess them are listed in Table 2. The full questionnaire battery took about 120 min to complete. A pilot data collection phase had indicated that the nature and length of questionnaire was appropriate for the target population. For those unable or unwilling to fill in the full questionnaire battery, a short version of the questionnaire was provided, covering physical and mental health outcomes and demographic information (an estimated 60 min to complete).

**TABLE 2:** Overview of the assessment instruments (Wave 3).

Format	Instrument (References)	Subconstruct
P&P	Instrument developed for the present study (n.a.)	Physical Health
		Physical Activity
		Sleep Patterns
		Nutrition
		Demographic Variables
		Biographical Cornerstones
		Evaluation of Study Participation
	Brief Symptom Checklist (Franke, 2017)	Psychiatric Symptoms
	NEO-Five Factor Inventory (Borkenau and Ostendorf, 2008)	Personality
	Cognitive Emotional Regulation Questionnaire (Loch et al., 2011)	Emotion Regulation
	Resilience Scale (Schumacher et al., 2004)	Resilience
	Health Questionnaire (Morfeld et al., 2011)	Subjective Physical and Psychological Functioning
	Pittsburgh Sleep Quality Index (Buysee et al., 1989)	Sleep Quality
	Satisfaction with Life Scale (Glaesmer et al., 2011)	Satisfaction with Life
	Sense of Coherence Scale (Schumacher et al., 2000)	Sense of Coherence
	General Self-Efficacy Scale (Beierlein et al., 2012)	Self-Efficacy
	Incongruence Questionnaire (Grosse Holtforth and Grawe, 2003)	Basic Psychological Needs
	Social Support Questionnaire (Fydrich et al., 2007)	Perceived Social Support
	Adult Attachment Scale – R (Schmidt et al., 2016)	Attachment Behavior
	Relationship Questionnaire (Bartholomew and Horowitz, 1991)	Attachment Style
	Retrospective 1-item measurement (Neumann, 2002)	Attachment to Own Parents
	Saarbrücken Personality Questionnaire (Paulus, 2011)	Empathy
NPA	Wechsler Adult Intelligence Scale (WAIS) (Petermann and Petermann, 2013)	Fluid Intelligence
		Crystalline Intelligence
		Verbal Intelligence
		Working Memory
		Numbers–Symbols Test
	Zurich Neuromotor Assessment II (Kakebeeke et al., 2018)	Fine and Gross Motor Skills
	Materialien und Normwerte für die neuropsychologische Diagnostik (Balzer et al., 2011)	Fluency
	Trail Making Test (Tombaugh, 2004)	Cognitive Flexibility
	Stroop Test (Strauss et al., 2006)	Inhibition
	Hopkins Verbal Learning Test (Brandt and Benedict, 2001)	Verbal Learning and Memory
	Rey Complex Figure Test (Rey, 1941)	Visual Construction, Figural Learning and Memory
	Instrument developed for the present study (n.a.)	Assessment-Related Health Issues
	n.a.	Body Weight and Height

In addition, the researchers invited participants to come to the Marie Meierhofer Children’s Institute in Zurich for neuropsychological assessment (estimated duration: 120–150 min). A full list of assessment instruments is listed in Table 2.

Questionnaire and test data were digitalized by entering it into digital questionnaires programmed with LimeSurvey (Limesurvey GmbH, 2020). This procedure facilitated data entry and reduced the potential for errors. Inter-rater reliability checks will be performed for test scoring. In addition, data entry was double checked by a second researcher for both test data and questionnaire data. Data was exported from LimeSurvey using a comma-separated format (.csv) and cleaned with R (R Core Team, 2018).

Preliminary power analyses indicated that for a *t*-test for independent samples with an alpha-error of 0.05 and a power of 0.80, at least 102 participants are required to detect medium effect sizes, while the detection of large effect sizes requires 42 participants.

Finally, participants were invited for biographical narrative interviews (Rosenthal, 1995), focused on the individual narrative that participants present when asked to talk about their lives. These interviews were conducted without a predefined time limit, and the participants are asked to explore the topics and share the experiences that are relevant to them as they wished. The interviews were conducted at the location of the participants’ choice. Interviews were recorded with an audio recorder and later transcribed and anonymized (Bohnsack et al., 2013). The audio files were then deleted to ensure data protection.

The time and nature of death of deceased participants was determined from the records of the municipal and civil registries.

#### Comparison With Zurich Longitudinal Studies

In the 1950s, Dr. Meierhofer was in close contact with Prof. Guido Fanconi, the medical director of the University Children’s Hospital who initiated the ZLS study in 1954. Dr. Meierhofer aligned the assessment instruments used to study the infants in the institutions with those used in the ZLS (Wehrle et al., 2020). Dr. Meierhofer studied the children at two time points: when children were between birth and three years of age (Wave 1) (Meierhofer and Keller, 1974), and a subsample of the children about 10 years later (Meierhofer and Hüttenmoser, unpublished). Children in the ZLS, were continuously assessed until age 18 (Wehrle et al., 2020). In a parallel effort, both cohorts are currently being located and assessed again. Once again, the study instruments have been closely aligned, thus allowing a 60-year longitudinal design with a comparison group.

#### Adjustments Related to the Pandemic of COVID-19

About midway through data collection for Wave 3, the COVID-19 pandemic reached Switzerland, and measures to contain the spread of the virus were implemented. Neuropsychological assessments and interviews on site were suspended for three months between mid-March and mid-June 2020 and then recommenced with protection measures. Data collection for questionnaire data continued throughout, and now includes a questionnaire to assess the impact of the measures to contain the pandemic on a range of outcomes: work and finances, home and social life, daily routines, and mental and physical well-being. This additional questionnaire was also sent to participants who had already completed data collection. Furthermore, this same questionnaire was sent to participants of the ZLS (for details see this issue Wehrle et al., 2020). This will allow us to check for any possible bias in data collected before or after the onset of the pandemic as well as whether reactions differed between the two cohorts to the measures implemented by the government.

### Data Analysis

Dr. Meierhofer and her team analyzed the historical data with the tools and the statistical techniques available at that time. For instance, tables and graphs were drawn by hand.

The historical data are now being re-analyzed with modern statistical methods to increase the reliability and validity of the historical results. Specifically, for data from Wave 1, we will seek to replicate Dr. Meierhofer’s finding that institutional placement and especially care conditions are significant predictors of the differences in children’s developmental outcomes. Our primary aim with the data of Wave 2 is to compare it with the data now available from the ZLS and to finally make the data available through publication.

Data will allow longitudinal analyses over two and three time points, both within group for children placed in institutions and between groups with children placed in institutions and children who grew up in families. The within-group analyses will examine the role of a set of potential predictors of inter-individual differences in growth in health and development. The between-group analyses will compare the two cohorts on the same outcomes.

We will apply structural equation modeling and multivariate regressions using a combination of variable- and person-centered statistical methods. These methods will allow us to account for measurement error (latent variable modeling), the multilevel structure of the data (multilevel modeling or correction of standard errors where multilevel modeling cannot be applied), and multiple covariates. A further important aspect of data analysis will be the implementation of multiple imputation methods to address missing data in general and selective drop-out in particular. A preliminary review of the raw data also indicates that historical data contains some errors that took place when transferring data between sheets or when rounding to the decimal. These errors will be corrected systematically.

For data from each wave, we will compute the deviation score of each participant of the cohort placed in institutions from the ZLS cohort mean. The resulting deviation score at Wave 3 will then be predicted by the deviation scores for Waves 1 and 2 by regression modeling. In addition, the influence of the individual variables on the longitudinal trajectory of health and development will be calculated using random-intercept autoregressive models (Hamaker et al., 2015) and latent growth models (Sticca and Perren, 2015). Multivariate associations among multiple constructs of interest will be examined using multivariate random-intercept cross-lagged models (Hamaker et al., 2015) and parallel process models (Sticca and Perren, 2015), depending on the research question at hand.

Qualitative data will be coded and descriptive analyses run and depicted in graphs where useful. Longer handwritten narratives and texts from Waves 1 and 2 will be subjected to content analysis (Mayring, 2000). Selected historical qualitative materials and narrative interviews conducted at Wave 3 will also undergo reconstructive and sequential analysis according to Rosenthal’s (Rosenthal, 2015) method. The aim of this abductive analysis method is to identify the latent content of the text, make generalizations, and come to conclusions via in-depth analyses of individual cases (Rosenthal, 2015).

Qualitative and quantitative data will be triangulated and historical data contextualized in the historic discourse on education and upbringing, structural violence, welfare practices, discrimination of certain family models, and compatibility of employment and family.

### Ethics

A number of measures identified ethical issues and mitigated risks for all three waves.

An independent ethics expert reviewed both historical studies (Wave 1: 1958–1961 and Wave 2 1971–1973), drawing on primary historical data and reports and publications. The review concluded that although research ethics practices have developed over time, Dr. Meierhofer adhered to the key ethical principles that hold today, chiefly the principle of not harming the subjects. The review even concluded that the children that took part in the study may have benefited; they certainly received some extra interaction time and attention as part of the study, in contrast to the deprivation prevalent in the institutions at that time. Assessment took place through observation and conversation and was non-invasive. At Wave 1, consent was provided by the head of the care institutions and at Wave 2 by the children’s legal guardians. Without such consent, children were not included in the study. Data was anonymized for analysis and publication (Brauer, 2019a).

For Wave 3, a number of ethical issues were identified. These included, for example, the risk of disclosing a previously unknown or not remembered institutional placement, the risk of disclosure of an institutional placement to next-of-kin or other third party, distress caused by learning about previously unknown inclusion in a study, discussing potentially distressing events from the past as part of data collection, and distress caused by accessing archived materials.

A comprehensive ethical framework and ethics protocol were developed that detailed every step of interaction with the individuals of the cohort with the aim of mitigating potential risks. In addition to detailed informed consent, voluntary participation, the option of withdrawing consent at any time, and protection of personal data, measures included a step-wise approach to contacting individuals with increasing amounts of information on the study, an option to claim mistaken identity, easy opt-out procedures, contact only with senior researchers, regular, standardized screening for distress, psychological support available on site for study participants, and psychological supervision available to researchers.

Furthermore, a thorough consent procedure was implemented, including seeking consent to link new and historical data. Participation was voluntary, and consent could be withdrawn at any time. Data will only be analyzed and published in an anonymized form.

These ethical considerations and ways to mitigate distress were developed and reviewed in consultation with an external, independent ethicist (Brauer, 2019b). Furthermore, findings from the exploratory study outlined in section “Exploratory Study” (Ryffel and Simoni, 2016) and the focus interviews conducted as part of the participatory study preparation (Lannen et al., 2020) (see section “Participatory research preparation”) also shaped the ethical framework of the study.

Wave 3 was reviewed and approved by the Ethics Committee of the Faculty of Philosophy at the University of Zurich (Approval Number 19.4.7).

## Discussion

A number of longitudinal studies over several decades on human development have been conducted in different parts of the world (for an exemplary overview see this issue Wehrle et al., 2020) and emerging data from robust longitudinal studies of children placed in institutions is becoming available (Nelson et al., 2014). However, the 60-year span of the LifeStories project presents the longest follow-up of children who spent their early years in an institution. Central to the feasibility of this study are a number of significant opportunities and challenges, with several lessons emerging from each of them.

### Historical Data

Comprehensive long-term longitudinal studies over several decades are rare, because they require continuous project leadership across several generations of researchers and an institutional home for the data to guarantee data access and data preservation over time. Other challenges of long-term longitudinal studies include the advancement of science overall: concepts and methodologies may change fundamentally after the initiation of the study. For instance, instruments used several decades ago might no longer be in use, thus making direct comparisons across the life span difficult. Some of these challenges are described and discussed in detail in the paper in this issue (Wehrle et al., 2020). For this study, even though it was dormant for over 40 years, access to data was possible through the continuity of institutional involvement and became accessible to the next generation of researchers at the Marie Meierhofer Children’s Institute. However, the fact that the data was not preserved in its entirety and had to be reassembled from a variety of documents—some organized by institution, some by subject, and some by outcome—posed a significant challenge. Using modern tools such as LimeSurvey templates and having another researcher enter data a second time mitigated the risk of data entry errors.

### Locating Individuals

A second challenge was to locate individuals after many decades while reducing possible false identification. However, being able to locate individuals through population registry is promising and has been successfully implemented, primarily by studies in the Nordic countries, where a central population registries exist (Kreicbergs et al., 2004; Lannen et al., 2008, 2010; Surkan et al., 2008; Lysholm and Lindahl, 2019). In Switzerland, the process is not centrally organized, and tracking individuals through the system over time proved to be very time consuming. Nonetheless, it was possible to find 86% (*n* = 268) of individuals as long as they still lived in Switzerland even decades after the previous contact. In fact, it proved possible to find individuals that Dr. Meierhofer was unable to locate at the time of Wave 2. It is plausible, but difficult to fully verify, that the reasons why individuals were not found in Switzerland included both death and a name change after an adoption.

Due to the substantial proportion of children of migrant workers (Meierhofer and Keller, 1974) in the cohort, almost one quarter of the individuals moved abroad. Finding these individuals is central to addressing some of the key research questions of the study, as the development trajectory of this subgroup of individuals might differ significantly due to familial backgrounds and reasons for placements. Tracing individuals who had moved abroad proved more difficult, but so far, 24% (*n* = 12) of eligible individuals living abroad were found.

### Contacting and Recruiting Study Participants

In recent years, a growing number of studies have examined the experience of formerly institutionalized individuals in Switzerland (Bombach et al., 2017, 2018b; Ammann et al., 2019; UEK Administrative Versorgungen, 2019). However, the primary approach of these studies was an opt-in approach in response to a call for contemporary witnesses to share their experiences. In contrast, because this was a pre-existing, predefined cohort, the study team approached a set cohort of individuals and invited them to continue their participation in the study after many decades. Although it is the availability of data from this cohort that provides the opportunity to conduct this longitudinal study, the study presents a number of challenges. Some individuals will be unaware of their former institutional placement, as they might not remember it due to their age at the time or due to repression of memories (Freyd, 1996; Ryffel and Simoni, 2016). This also might be a period of their lives that was not discussed in the family because it was considered stigma or taboo (Sack and Ebbinghaus, 2012; Ryffel and Simoni, 2016; Lengwiler, 2018). Hence, contacting them might disclose this information. A number of measures were put in place to mitigate some of the risks when contacting the individuals, including a comprehensive ethical framework and ethics protocol and psychological support available on site. Furthermore, all contact with the individuals was made solely by senior staff members. This proved essential when having to respond to diverse biographical themes triggered during contact procedures. For example, as a result of their past experiences with authorities, concerns about being purposely misled or judged by the researcher surfaced regularly (Lannen et al., 2020). Working with senior researchers was also key to responding appropriately to any questions, concerns, or potential distress that arose during contact. Earning the trust of the participants was essential. This was achieved chiefly by consistency; the same researcher stayed in contact with the individual throughout the study. It was also achieved by the researchers’ efforts to be empathetic no matter what situations came up and to be “humanly available” to the participants (Lannen et al., 2020). Trust was further built by the ability within the research team to deploy researchers with the same linguistic backgrounds as the study participants (Swiss-German, German, Italian, Spanish).

Another important element proved to be the participatory approach to study preparation that included individuals affected by institutionalization as children. Their input significantly shaped the narrative, the approach of the study, and the researchers’ skills (Lannen et al., 2020). It also provided an opportunity to respond to the request of those affected to be included in research related to the historic reappraisal process of compulsory social measures and placements before 1981 in Switzerland (UEK Administrative Versorgungen, 2019).

Finally, the project built on Antonovsky (1987) and Werner (2013) findings and a belief that the life trajectory of an individual is determined by many factors of internal and external nature. When asked to provide a recommendation on what would be helpful to formerly institutionalized children in overcoming their experience, Gahleitner, as part of her expert mandate in the round-table discussions related to institutional placement in Germany, stated: “A salutogenetic approach (…) allows insights that are usually lost when the focus lies on a search for deficits”^11^ (Gahleitner, 2009).

This was expressed in how letters to the participants were formulated and how researchers interacted with them. It was also expressed in the outcome measures included in data collection and an attempt to move away from a solely deficit-oriented narrative in relation to their starting conditions. While prepared for any distress expressed by the participants as a result of past events, the team was also able to acknowledge evidence of resilience and a sense of coherence (Antonovsky, 1987). Overall, the approach attempts to overcome the risk of categorizing the individuals in the group of individuals that started unfavorably and developed poorly (those in infant care) compared to a community sample of individuals that grew up at home and therefore are considered to be the norm (ZLS). The study is embedded in a framework of respect and empathy for each individual’s life story. It is open to a multitude of life trajectories between and within each study arm without prejudging the outcomes. At the same time, the framework honors the sense of injustice felt by many of the individuals subjected to welfare practices implemented before 1981. It is an attempt to honor their experiences without passing judgment, and also seek the potential resilience and strength that may have grown from each individual’s unique experience.

### Methodological Approach

A number of factors fundamentally shaped the methodological approach of the study. These included the feedback on instruments from individuals affected by institutional placement as children during study preparation. Moreover, the research team applied a multidisciplinary and multimethod approach. Leveraging backgrounds in developmental psychology, pediatrics, educational sciences, sociology, and anthropology allowed the team to use validated instruments for assessment of health as well as psychological constructs and combine them with, for example, reconstructive methods. Although this approach required constant discussions and feedback loops in order to negotiate common terminology and framing, it enabled a holistic view of the interplay between individual development and a particular societal and educational context. Furthermore, the expertise combined in quantitative and qualitative approaches enabled the collection of both standardized and narrative data and thus reflect the complexity of the human experience as completely as possible.

However, this study also faced a number of key methodological challenges:

First, the availability of a community sample of individuals that grew up in the same geographic location at the same time (Wehrle et al., 2020) enables us to distinguish historical variables from biological, personal, and social ones and extract generalizable statements and recommendations. However, children placed in institutions in the late 1950s were exposed to multiple risks: both the societal, legal, and familial circumstances that put the children at risk of institutionalization in the first place and the deprivation the children experienced as part of the institutional placement (Meierhofer and Keller, 1974). This is relevant, because other studies have found evidence of a dose–response effect in that the more adverse events a child is exposed to, the more profound and lasting the effect on the individual will be (Felitti et al., 1998; Finkelhor et al., 2007; Huebner et al., 2016). As part of this study, it will be important to distinguish pre-existing risks as much as possible from the impact of institutionalization and possible compensatory effects arising from relationship conditions with parents or better institution quality by including variables in our models that account for possible additive and interaction effects. Fortunately for us, children were generally assigned to institutions independently of variables such as health, development, or potential covariates such as family background. Placement was decided by geographic location or space availability, hence mirroring a quasi-experimental design of children with similar distribution of backgrounds and preconditions in each institution and thus increasing the probability that differences between development outcomes in the different institutions are due to circumstances in the care facility.

Second, individuals in this cohort are organized by institution in 12 clusters. This is a challenge, as 12 clusters is at the very low end, if not below the required amount of clusters from a statistical point of view. The other challenge arises when comparing data with the ZLS, as this multilevel structure of data is only present in one cohort. In addition, some information only exists for the entire institution even though it can be assumed that conditions within the institutions varied between units within a single institution. Combined expertise in the project team, the project board and through collaboration with specific experts will enable us to address this methodological challenge through multilevel modeling or correction of standard errors where multilevel modeling cannot be applied.

Third, there are a number of challenges inherent to longitudinal studies that run for several research generations. These challenges are described in detail in the paper of this issue (Wehrle et al., 2020), and include dealing with archived data and their sometimes spotty documentation, the predefined nature of the cohort or balancing continuity of measurements while keeping up with latest standards.

Fourth, the more time passes, the greater grows the chance of bias as a result of the possible impact of the events on mortality for those more severely affected, as described in seminal literature (Felitti et al., 1998). Furthermore, the results of the preliminary study (Ryffel and Simoni, 2016) also suggest that a self-selection bias might have operated toward participants with more adaptive long-term outcomes. In addition, women and the children of single mothers were over-represented compared to migrant families. Analyzing whether participation in the study is selective in relation to key variables from Wave 1 and 2 is essential.

### Research Questions

Mastering these challenges will allow us to address a number of research questions.

Historical data:

(1) Circumstances of placements:
  What information can be found on why infants were institutionalized as part of historic welfare practices, and how is this represented in the literature on relevant discourses from that time? What were the conditions and routines like within the institutions?
(2) Health and development of children
  Can findings from the historical data be replicated? What additional findings related to the impact of infant institutionalization can be brought to light from the historical data? What additional and relevant insights can be found by comparing Wave 2 to the comparison group of the ZLS? How do familial and institutional contexts relate to children’s health outcomes and abilities?

What deficits identified in Wave 1 and Wave 2 can still be detected in individuals 60 years after they were institutionalized as infants?

Newly collected and longitudinal data:

How have the lives of individuals that spent their infancy in institutions evolved? What is their morbidity (physical and mental) and mortality? What are their cognitive, social, and motor abilities today? What are their educational paths and socio-economic conditions? What deficits identified in Waves 1 and 2 can still be detected in individuals 60 years after they were institutionalized as infants? What specific vulnerabilities and/or strengths have emerged during the life course? How have some of the care circumstances and practices related to institutional placement affected long-term outcomes? What are the key individual variables and life events that moderate the relationship between early institutionalization and long-term outcomes? How do individuals talk about their life trajectories? What themes are relevant to them and how do they make sense of what happened?

### Outlook and Conclusion

Overall, the study will allow us to better understand the effects of being placed in infant institutions under conditions of deprivation, will improve our knowledge of possible development trajectories, and will contribute to the reconciliation process and historical reappraisal process of compulsory social measures and placements before 1981 in Switzerland. It will reveal important insights for children placed in institutions today and allow us to better understand how children with different starting conditions can be supported in developing healthily. Finally, the present study will provide a unique contribution to our understanding of the interplay between individual and environmental promotive and protective factors for development over decades in individuals at risk.

A number of opportunities exist when considering potential outlooks for the study. The individuals of the LifeStories project are now at the cusp of old age. Some evidence has recently emerged demonstrating that exposure to adverse events in childhood is linked to premature biological aging in adulthood (Shalev et al., 2013; Puterman et al., 2016). However, this data was collected retrospectively. Longitudinal data is needed on how adverse events may affect biological aging.

Consent from all participants of Wave 3 was sought to contact them again in the future for additional prospective assessments. Thus, collecting biological and neuroimaging data will be important to further understanding the specific aging processes in this vulnerable cohort.

In addition, collecting information from additional perspectives will allow us to complement the efforts related to the historic reappraisal process of compulsory social measures and placements before 1981 in Switzerland. To better understand the circumstances that led to infant care placements at the time, the perspective of the parents of formerly institutionalized infants would be essential and is time sensitive due to their advanced age. This also applies to former employees of the institutions. In addition, interviews with children of those placed in institutions are relevant, as emerging evidence shows the effect such measures may have from a generational perspective (Bombach et al., 2018a,b; UEK Administrative Versorgungen, 2019).

Making the details of the study process available as part of a study protocol allows the scientific community to learn what works to successfully conduct longitudinal studies and shed light onto the secrets of long-term adaptation processes across the lifespan.

## Dissemination

The results bear significant potential for scientific, practical, and social impact at national and international levels, and results will be disseminated accordingly.

Understanding the impact that measures related to compulsory social measures and placements before 1981 in Switzerland had on the children during infancy and childhood and the consequences 60 years later will contribute to the historical reappraisal and political reconciliation of such measures in Switzerland. Accessing historical records will enable a data-driven approach to understanding the causes, characteristics, and mechanisms that led to and surrounded infant care practices before 1981. It will allow society to make sense of how practices came about and enable it to uncover the zeitgeist and societal norms and values that framed the actions. The study will illuminate a piece of Swiss institutional history, provide a basis to reflect on today’s practice critically, and might sensitize the society to the possible existence of blind spots in today’s practices in Switzerland and around the world.

Due to its longitudinal design, the study is highly relevant and one of the few long-term follow-up studies of its kind. Through the existence of a comparison group from the same era (Wehrle et al., 2020), historical variables can be distinguished from biological, personal, and social ones, and generalizable statements and recommendations can be extracted that are relevant for today. This will improve our understanding of the detection and handling of difficult early life conditions and our support of the development of resiliency over a life-course perspective.

It will also improve our understanding of the long-term consequences of deprivation caused by institutionalization of young children. The study will reveal factors mitigating institutional care and protective processes for favorable development trajectories as a result of resiliency processes. It will also provide information on how the professional and policy community can best support children and young people in care and their families across the globe.

To this end, scientific publications focused on the key research questions will be published in peer-reviewed journals related to medicine, psychology and educational and other social sciences, and results will be presented at international scientific conferences of these disciplines. In addition, a significant effort will be made to make results accessible to a non-scientific expert audience, society and the survivors of compulsory social measures and placements before 1981.

Evidence briefs will serve as a key tool for disseminating results to a non-scientific audience. A set of evidence briefs will be published in German with a local focus related to the reappraisal and rehabilitation of compulsory social measures and placements before 1981. Additional briefs that focus on the results of international relevance will be published in German and English and target professionals and key organizations involved in out-of-family placements and a multidisciplinary audience of professionals and policy-makers working with individuals at all stages of life. Further, we will work toward a book publication with some of the historical images and materials available. In addition, roundtable discussions will be held with relevant professionals. Results will be presented at local conferences for a non-scientific audience. Efforts will be made to develop a curriculum that includes our findings in university-level training (psychology, social works, educational sciences) and with children in primary and secondary school settings.

All publications will be made available free of charge to participants if desired.

## Ethics Statement

The studies involving human participants were reviewed and approved by Ethics Committee of the Faculty of Philosophy at the University of Zurich. The patients/participants provided their written informed consent to participate in this study.

## Author Contributions

PL is the principal investigator of the study. She drafted the Abstract, Introduction, several sections of the description of Wave 3 (Study Preparations, Eligibility, Comparison with ZLS, Adjustments to COVID-19, and Ethics), the Discussion, and the Dissemination section. She edited all other sections of the manuscript. HSa is completing her doctoral work as part of this study. She prepared the historical data and drafted the section on Design and Analysis for Wave 1 and 2. She also contributed significantly to the section on locating individuals. She provided input to the Discussion section and the section on data collection of Wave 3. FS is a senior researcher in the project and drafted the section on data collection and analysis of Wave 3. He provided input to the discussion section, in particular the section on methodological challenges. IRG is a research assistant in the project. He drafted the section on locating individuals and provided input to the discussion section and the section on contact procedures. CB is a senior researcher in the project. She drafted the section on contact procedures and edited the manuscript. In particular, she provided input to the sections on Study Preparation, Ethics, Discussion, and Dissemination. HSi is a co-investigator of the study. She provided input to the Introduction and Discussion sections and edited a final version of the manuscript. FW is a senior researcher in the Zurich Longitudinal Studies (ZLS). She provided input to the sections about the ZLS and the Discussion section. OJ is a co-investigator of the study, provided input to the sections about the ZLS and the Introduction and Discussion sections, and edited the final version of the manuscript. He is the principal investigator of the Zurich Longitudinal Studies. All the authors contributed to the article and approved the submitted version.

## Conflict of Interest

The authors declare that the research was conducted in the absence of any commercial or financial relationships that could be construed as a potential conflict of interest. The reviewer MM declared a shared affiliation, though no other collaboration with the authors to the handling editor.

## Footnotes

1 In the further course of the study, Dr. Meierhofer excluded additional children because they either had organic disorders only diagnosed in the further course of the study, their data collection could not be completed (e.g., because information on family’s social background was not available), or because a further examination of administrative records revealed that the children did not, after all, meet the selection criteria concerning age at in-care placement or continuity of the institutional stay. Some children were excluded because, due to an intermediate change of institution, they were accidently tested twice. Thus, the final sample for Dr. Meierhofer’s analysis consisted of 391 children.

2 As part of a diploma thesis, Ingrid Meyer-Schell conducted a preliminary follow-up study between 1969 and 1971. From the total sample of the first assessment wave, she only selected children of Swiss nationality, born to unmarried mothers, and who were younger than 33 months at the first assessment wave. They also had to be between 11 and 12 years old at the time of the follow-up study. Of 45 children contacted, 16 children were allowed to participate. Standardized tests to assess intelligence (Stanford-Binet Intelligence Test, Terman and Merrill, 1965, and Goodenough-Draw-a-Man Test, Goodenough, 1926), frustration tolerance (Rosenzweig Frustration Test, Duhm and Hansen, 1957) and conflicts and motivations (Sceno Test, von Staabs, 1964) were used as well as additional questionnaires to include children’s, parents’, and teachers’ perspectives.

3 For some children, it was even possible to obtain basic information from administrative records or through parents or caregivers during telephone contact, even if the children did not participate in the follow-up study.

4 Each resident in Switzerland with Swiss Nationality also has a “commune of origin” (*Heimatort*), similar in function to “place of birth” in other countries. The office of civil registry (*Zivilstandsamt*) in that commune of origin holds key documents such as birth certificates and marriage records. The civil registry records the place of residence at the time of events relevant to civil status (such as marriage, divorce, change of name).

5 Cantons are the member states of the Swiss Confederation.

6 In the Canton of Zurich, for example, this is stated in §18 section. 1 Law on registration and population registers (German: *Gesetz über das Meldewesen und die Einwohnerregister, MERG*).

7 In the Canton of Zurich, for example, this would be *Bekanntgabe von erweiterten Personalien*, §18 Section 2 MERG.

8 Canton of Zurich: §23 Law on information and data protection (German: *Gesetz über die Information und den Datenschutz, IDG*).

9 Canton of Zurich: §22 IDG.

10 Canton of Zurich: §19 IDV.

11 Original in German: “*Die salutogenetische Orientierung* (…) *bietet Einsichten, die in der reinen Suche nach Defiziten häufig verloren gehen*.”
